# A Review of Pathogens, Diseases, and Contaminants of Muskrats (*Ondatra zibethicus*) in North America

**DOI:** 10.3389/fvets.2020.00233

**Published:** 2020-05-15

**Authors:** Laken S. Ganoe, Justin D. Brown, Michael J. Yabsley, Matthew J. Lovallo, W. David Walter

**Affiliations:** ^1^Pennsylvania Cooperative Fish & Wildlife Research Unit, Department of Ecosystem Science and Management, The Pennsylvania State University, University Park, PA, United States; ^2^Department of Veterinary and Biomedical Sciences, The Pennsylvania State University, University Park, PA, United States; ^3^Southeastern Cooperative Wildlife Disease Study, Department of Population Health, College of Veterinary Medicine, University of Georgia, Athens, GA, United States; ^4^Warnell School of Forestry and Natural Resources, University of Georgia, Athens, GA, United States; ^5^Bureau of Wildlife Management, Pennsylvania Game Commission, Harrisburg, PA, United States; ^6^U.S. Geological Survey, Pennsylvania Cooperative Fish and Wildlife Research Unit, The Pennsylvania State University, University Park, PA, United States

**Keywords:** *Ondatra zibethicus*, North America, population health, parasites, heavy metals, agricultural contaminants, viruses, bacteria

## Abstract

Over the last 50 years, significant muskrat (*Ondatra zibethicus*) harvest declines have been observed throughout North America. Several theories for the decline have been proposed, including increased parasite infections and disease within muskrat populations. No existing wholistic review of muskrat exposure to pathogens, contaminants, and diseases exists. To address this knowledge gap, we conducted a thorough review of existing literature on muskrat pathogens, contaminants, and diseases across their natural range. This review is comprised of 131 articles from 1915 to 2019 and from 27 U.S. states and 9 Canadian provinces. A wide diversity of contaminants, toxins, and pathogens were reported in muskrats, with the most common diseases being cysticercosis, tularemia, Tyzzer's disease, and biotoxin poisoning from cyanobacteria. This review provides a summary of muskrat pathogens, contaminants, and diseases over a century that has observed significant population declines throughout the species' range in North America. Such data provide a baseline for understanding the potential role of disease in these declines. In addition, these data highlight critical knowledge gaps that warrant future research efforts.

## Introduction

The muskrat (*Ondatra zibethicus*) is a wide-spread furbearer species in North America (1). Since 1970, muskrat harvest estimates have declined in the northeastern U.S (2). Evidence of declines in muskrat harvest has also been observed throughout the native range of muskrats, with decreases exceeding 50% in some states (3). Harvest estimates have historically been used in combination with other methods to estimate game species population abundance in order to adjust bag-limits on harvest during sequential years (4).

The observed muskrat harvest decline suggests a population decline across much of North America. Several theories for the widespread muskrat declines have been proposed, including habitat loss, increased flooding events, predation, and environmental contamination (3). In addition, other ancillary factors, including infectious and non-infectious diseases, have been suggested as contributing to the observed declines (5).

Muskrats are a semiaquatic species that thrive in a variety of habitats, including marshes, ponds, streams, and rivers. Consequently, muskrats are potentially exposed to a high diversity of pathogens and contaminants, including those associated with terrestrial and aquatic ecosystems. For example, muskrats have reportedly been infested with mites commonly found on terrestrial mammals (e.g., *Listrophorus* and *Laelaps* spp.) as well as with water mites (*Hydrachnidia* spp.) (6). In addition, because muskrats have a wide geographic range throughout North America, regional differences in pathogen and contaminant exposures may occur.

While there is abundant literature on pathogen and contaminant exposure of muskrats in North America (Table 1), existing data are insufficient to evaluate whether infectious or non-infectious diseases are contributing to the observed declines. Most of the comprehensive reviews to date have been regionally specific and/or conducted on data from 1914 to 1948, which predates the observed declines (Table 2). In addition, existing reviews have focused exclusively on parasites and did not include non-infectious diseases (e.g., contaminants and toxins) or infectious diseases requiring contemporary diagnostics (e.g., bacteria, fungi, and viruses). Consequently, an extensive review that incorporates data on all pathogens and contaminants of North American muskrats was warranted.

**Table 1:** Number of species reported of each respective parasite category in historic literature reviews of muskrat (*Ondatra zibethicus*) parasites from 1947–1986.

	**Musfeldt (7)**	**Meyer and Reilly (8)**	**Knight (9)**	**Beckett and Gallicchio (10)**	**Kennedy (11)**
Protozoa	4	0	4	0	4
Trematoda	26	29	27	18	22
Cestoda	9	8	9	8	11
Nematoda	13	12	14	7	8
Acarina	4	5	6	0	0
Pentastoma	0	1	0	0	0
Arachnida	1	0	1	0	0
Acanthocephala	0	0	0	2	1
Insecta	0	0	1	0	0
Total	57	55	62	35	46

**Table 2:** Range of years, number of studies cited, and geographical representation covered in respective historical review articles on muskrat (*Ondatra zibethicus*) parasitology.

**Author**	**Years reviewed**	**Studies cited**	**Geographical representation**
Musfeldt (7)	1914–1946	32	British columbia
Meyer and Reilly (8)	1909–1949	38	Maine
Knight (9)	1914–1948	34	British columbia
Beckett and Gallicchio (10)	1951–1966	19	Ohio
Kennedy (11)	1930–1981	25	Canada

The objective of this study was to review existing peer-reviewed data and technical reports on pathogen (virus, bacteria, fungi, parasites) and contaminant exposure of North American muskrats, with an emphasis on those causing morbidity and mortality. Unfortunately, there have been significant taxonomic changes for many of the pathogens over the time period covered in this review. This has resulted in some parasite identifications being unreliable due to lack of detail in the original data or taxonomic revisions (i.e., splitting of species), so these taxonomic changes are highlighted and explained throughout the manuscript.

## Materials and Methods

Existing literature related to exposure of muskrat in North America to pathogens or contaminants was obtained from Google Scholar and Web of Science ™ using a combination of keywords, including muskrat, infection, disease, contaminant, parasite, health, and exposure, as well as several other more specific pathogen and contaminant names. Sources referenced within literature found using the search engines were also investigated for additional relevant publications. Information collected from articles (if applicable) included: year of survey, location of survey, methodology, number of animals surveyed, pathogen/contaminant identity, presence or absence of associated disease (as evidenced by reported clinical signs or lesions), prevalence, and other pertinent information. Data were split into sections based on the pathogen or contaminant identity (e.g., viruses, bacteria, parasites, and toxins and contaminants) and all relevant data listed above were summarized. Prevalence values and ranges reported in this manuscript reflect the sample prevalence reported in the respective studies. Sampling effort varies between studies and affects detection rates, consequently, it should be noted that true prevalence may not accurately represented by the prevalence reported.

## Results

### Viruses

**Figure 1:**
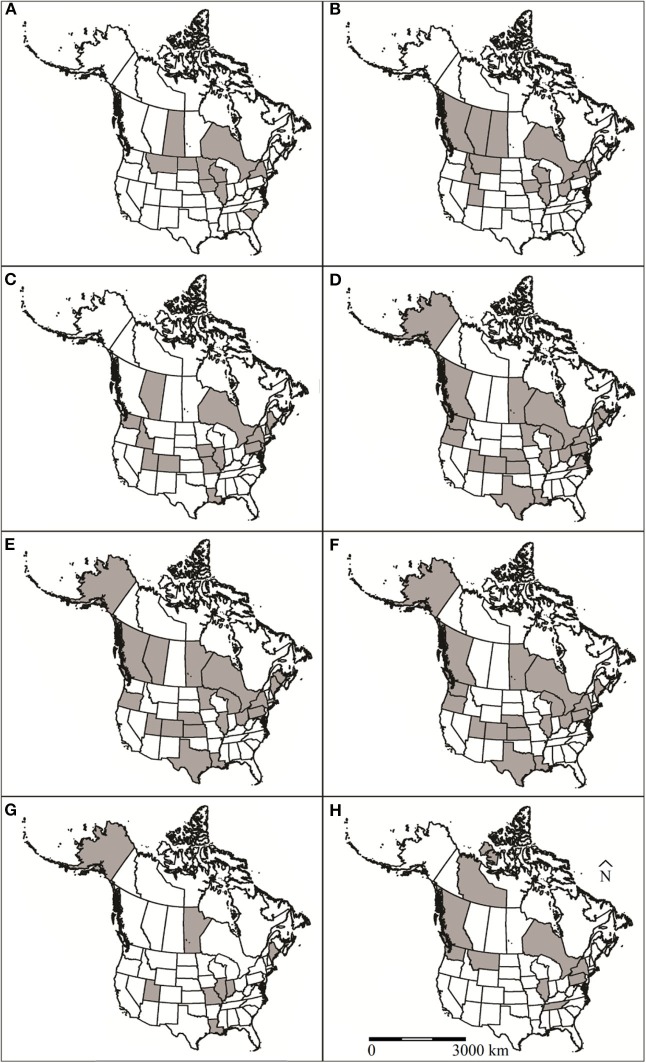
U.S. states and Canadian provinces that muskrat (*Ondatra zibethicus*) health surveys have been conducted in North America for **(A)** viruses, **(B)** bacteria, **(C)** protozoan parasites, **(D)** trematodes, **(E)** cestodes, **(F)** nematodes, **(G)** ectoparasites, and **(H)** toxins and contaminants. Locations designated by shaded regions.

Exposure to or infection with viral pathogens have been reported in 14 papers representing four U.S. states and three Canadian provinces (Figure 1A). All of these papers were recent, relative to reports of other etiology and occurred between 1966 and 2017. Six different viruses have been screened for in muskrats, including canine distemper virus, rabies, *Orthohepevirus*, Aleutian mink disease virus, and adenovirus (Table S1). The most commonly reported virus screened for was rabies virus (*n* = 9 reports). Five of these rabies reports were from passive rabies virus surveillance conducted by state agencies that were reported to the Centers for Disease Control and Prevention and the remaining four were post-mortem examinations by research groups. Thirteen muskrat mortalities have reportedly been associated with rabies virus infection (Table S1). Antibodies to canine distemper virus (*Morbillivirus*), and *Orthohepevirus* have been detected in muskrats during serosurveys but have not been reportedly associated with morbidity or mortality (12, 13). Thirteen cases of rabies virus were detected in muskrats in the U.S. in several studies, with a concentration of detections along the border of the U.S. and Canada (Figure 1A) (Table S1).

### Bacteria

Based on their habitat utilization, muskrats are frequently exposed to virulent and avirulent bacteria. Bacterial infection can occur directly (i.e., direct contact with conspecifics), vertically (i.e., *in utero*), or indirectly through bacteria in the environment (i.e., contaminated water) (14). The clinical outcome of infection can vary dramatically between host species and pathogens, with even significant bacterial diseases (e.g., cholera, tularemia, and plague) presenting with a spectrum of clinical signs and lesions.

Since 1952, 24 species of bacteria were reported in muskrats in 23 publications representing 10 U.S. states and five Canadian provinces (Figure 1B). The bacterial infections were associated with morbidity/mortality in 16 of these publications (Table S1). The most common species of bacteria reported in muskrats were *Francisella tularensis* (*n* = 11) and *Clostridium piliformis* (*n* = 5). Five of the bacterial species reported from muskrats were associated with morbidity/mortality, including *F. tularensis, Francisella philomiragia, C. piliformis, Staphylococcus* sp., and *Anabaena flos-aquae*. *F. tularensis, F. philomiragia, C. piliformis, and Staphylococcus* sp. cause disease via invading and destroying tissues whereas *A. flos-aquae* produce exotoxins that are ingested by the host (15, 16).

#### Tularemia

Tularemia is caused by *F. tularensis*, which has both terrestrial and aquatic cycles. The two subspecies, *F. t. tularensis* and *F. t. holartica*, are referred to as type A and type B, respectively. Type B occurs globally throughout the Northern Hemisphere and southern Australia, whereas type A occurs only in North America. The two subpopulations of type A are type AI and type AII, and they are found generally east and west of the 100th meridian, respectively (17). Subpopulation type AI is more pathogenic and mostly infects terrestrial mammals (18). While hundreds of animal species can be infected with *F. tularensis*, rodents and lagomorphs are the main terrestrial hosts (19). Muskrats and beavers (*Castor canadensis*) serve as the primary hosts in the aquatic cycle and are most commonly infected with the less virulent type B. However, epizootics of tularemia in muskrats have been reported in northern North America, specifically Alberta and Ontario, Canada and Vermont, U.S. (Table S1). These outbreaks are commonly associated with aquatic habitats such as streams and marshes. Outbreaks are also thought to be related to increased prevalence of the bacteria in reservoirs such as voles (*Microtus* spp.). Voles carry the less virulent type B and excrete bacteria in large numbers in their urine (19). *F. tularensis* can be carried by a variety of animals or insects such as rodents, ticks, fleas, and mosquitos (20). Humans and other animals can become infected with the bacteria by ingesting contaminated food and water, breathing contaminated air, or most commonly being bitten by a vector (e.g., ticks) or through direct contact with wildlife, especially lagomorphs and small rodents (21).

Although there are many cases of tularemia in humans and wildlife, little is known about the life cycle and persistence of *F. tularensis*. However, outbreaks in sheep and humans have coincided with epizootics in rodents and lagomorphs suggesting transmission from either the latter to humans and sheep or through vectors such as flies or ticks (20). Pathological changes vary depending on the type of infecting *F. tularensis*, however in muskrats the most common is lesions on the liver (22). For muskrats, six of the 11 articles report mortality due to *F. tularensis* infection (Table S1). The majority of these reported deaths occurred in Canada (Table S1). Monitoring tularemia outbreaks in not only muskrats, but also other animals and humans, can aid in the understanding of the transmission patterns of this disease.

#### Other Bacteria and Fungi

Both *F. philomiragia* and *Staphylococcus* sp. have reportedly been associated with mortality in individual muskrats; however, confirmatory diagnostic tests in many of these cases were questionable. *F. philomiragia* (formerly, *Yersinia philomiragia*) was isolated in a muskrat carcass with hepatized lungs in Utah in 1969 (23). A single muskrat found dead in Illinois was screened for a variety of diseases and parasites and the cause of death was determined to be an infection of *Staphylococcus* sp. This muskrat also had a secondary viral infection (24). A case of Psittacosis related to undetermined *Chlamydia* spp. resulted in mortality of two muskrats from Saskatchewan, Canada. Upon examination, 14% of muskrats collected were positive for the bacteria (25). No further reports of *Chlamydia* spp. have been documented in muskrats in North America. Three species of fungi, *Emmonsia crescens, Encephalitozoon cuniculi*, and *Trichophyton mentagrophytes*, were reported in muskrats from Utah, Saskatchewan, and Iowa, respectively (26–28). However, prevalence of fungal infection was low (range: 2.94–7.69%) and has not been documented in muskrats since 1979 (27).

#### Tyzzer's Disease

Tyzzer's disease is an acute bacterial disease caused by *C. piliformis* (formerly *Bacillus piliformis*) (29). The disease has been reported in a variety of species, including raccoon (*Procyon lotor*), mice (*Mus* spp.), coyote (*Canis latrans*) and cottontail rabbits (*Sylvilagus* spp.) (30–32). Outbreaks of Tyzzer's disease are acute and commonly associated with increased stress due to factors such as changing environmental conditions or secondary infections compromising immune function (33). Individuals infected with *C. piliformis* shed more spores when stressed, leading to increased environmental contamination (34). The spores remain infectious in the environment (e.g., inside contaminated muskrat huts) for at least 5 years, allowing the reinfection of muskrats re-colonizing abandoned huts and burrows (35). During most outbreaks, muskrats were found dead without premonitory signs, however, clinical signs and lesions included hemorrhagic enteritis and liver lesions (29, 33). Of note, the disease was historically separated between Errington's disease and Tyzzer's disease, with the causative bacteria identified as *Clostridium* sp. and *B. piliformis*, respectively. There is much support in the scientific community that Errington's and Tyzzer's are the same disease, therefore Tyzzer's disease has become the adoptive name (36, 37). Mortality from Tyzzer's disease in muskrats has been reported in six studies from three U.S. states and two Canadian provinces in the years 1966, 1971, 1977-79, and 2019 (Table S1). Of studies that reported sample size (*n* = 4 studies), a total of 67% mortality was reported among the studies (Table S1).

#### Cyanobacteria

One species of cyanobacteria (*A. flos-aquae*) has caused mortalities in muskrats (Table S1). Cyanobacteria (i.e., blue-green algae) can form extensive algal blooms on the surface of water and produce toxins. There are two main categories of toxin produced by cyanobacteria, cytotoxins and biotoxins. Cytotoxins are not severely harmful to living organisms that ingest them; however, biotoxins can be (38). Wildlife and domesticated animal deaths have been attributed to biotoxin poisoning from animals drinking water with planktonic cyanobacteria floating on the surface (39, 40). In the U.S., 18 muskrats along with other wildlife species were found dead in Iowa lakes during the fall of 1952 (16). These lakes were sites of *A. flos-aquae* blooms, and researchers attributed the wildlife mortality to cyanobacterial toxins (16). Clinical signs of cyanobacterial poisoning ranged from anorexia and diarrhea to hypersalivation and convulsions (38). Cyanobacterial poisoning of muskrats can cause mortality directly or through bioaccumulation in filter feeding bivalves (e.g., mussels). In lakes of Alberta, clams displayed the ability to accumulate microcystin-LR (MC-LR), a toxin produced by cyanobacteria. *Microcystis aeruginosa* was the main producer of MC-LR in the study areas (41). MC-LR's ability to bioaccumulate and pass along the trophic level introduces a level of concern for the possible poisoning of higher trophic organisms.

### Parasites

Parasites can exert negative impacts on the health of their hosts through a diversity of mechanisms, including direct morbidity or mortality due to tissue damage or indirectly by utilizing host resources, decreased growth and survival of young, or altering host susceptibility to other pathogens (42–44). Several taxa of parasites have been reported from muskrats in North America, either sporadically or commonly, including protozoans, trematodes, cestodes, nematodes, acanthocephalans, pentastomes, and ectoparasites (Table S1). Discussion of individual parasites in the text are restricted to those which have been reported in more than 10 individual hosts or are associated with disease.

#### Protozoa

Protozoa are single-celled eukaryotes that vary in pathogenicity depending on the parasite species and host susceptibility (45, 46). Protozoan life cycles may be complex and require different host species for development. A number of protozoa have wide host ranges which include not only wildlife species, but also humans and domestic animals (47).

Since 1936, 18 articles have reported eight protozoan species in muskrats. These studies represent samples from 17 U.S. states and all of Canada (Figure 1C). Most cases in the U.S. were from three geographic regions: Snake/Colorado River Drainage Areas, Mississippi River Drainage, and Northeastern states (Figure 1C). The most commonly reported protozoa included *Giardia* spp. (*n* = 10 studies), *Toxoplasma gondii* (*n* = 3 studies), and *Cryptosporidium* spp. (*n* = 3 studies). Other species of intestinal protozoan parasites documented in muskrat were *Chilomastix* sp. (*n* = 1 study), *Eimeria* spp. (*n* = 2 studies), *Sarcocystis jaypeedubeyi* (*n* = 1), and *Trichomonas* sp. (*n* = 2 studies).

The protozoan species and their observed prevalence in muskrats varied among studies. *Giardia* spp. were reported in ten studies representing 14 U.S. states and Alberta with prevalence ranging from 36 to 100% (Table S1). *Cryptosporidium* spp. were reported in three studies representing four states in addition to Alberta with prevalence ranging from 0 to 50% (Table S1). While these parasites do not appear to have significant health impacts for muskrats, some may have health implications for humans and domestic animals (e.g., *Giardia* and *Cryptosporidium*) depending on the genotype infecting the muskrats (48, 49).

A study in Maryland examined 1,581 muskrats and attributed two mortality events to intestinal coccidiosis, but the etiologic agent was not determined (50). Another study in Canada determined that *Eimeria ondatrazibethicae* (reported as *E. stiedae*) was the cause of liver coccidiosis in the muskrats (51). Both *Giardia* spp. and *T. gondii* are protozoan parasites that can cause serious disease in humans and some domestic and wild animals [e.g., sea otters (*Enhydra lutris*)] (52) and although muskrats are commonly infected with these parasites, disease has not been reported in them (Table S1).

#### Phylum Platyhelminthes: Class Trematoda

Trematodes (i.e., flukes) are endoparasites transmitted to mammalian hosts by either ingesting the intermediate host (e.g., snails), or by coming in contact with the free-swimming ciliated larva that then penetrates the skin of the host (53, 54). Diseases such as fascioliasis, echinostomiasis, and schistosomiasis are caused by trematodes or their larval forms and occur in a variety of mammalian species (55–57).

Since 1915, 46 articles have reported 32 species of trematodes in muskrat from 19 U.S. states and six Canadian provinces (58). The geographical distribution of these reports is widespread with no obvious spatial pattern (Figure 1D). The most commonly reported species are intestinal flukes: *Echinostoma revolutum* (*n* = 28 studies), *Quinqueserialis quinqueserialis* (*n* = 27 studies), *Notocotyle urbanensis* (*n* = 18 studies), *Plagiorchis proximus* (*n* = 18 studies), and *Wardius zibethicus* (*n* = 17 studies) (Table S1). Since 1938, seven articles have reported blood flukes (*Schistosoma* sp.) in muskrats. There is only a single report of a lung fluke (*Paragonimus* sp.) in a muskrat (59).

The prevalence and burdens of individual trematode species was highly variable. For example, the prevalence of *Echinoparyphium* sp. in four provinces and five U.S. states was relatively low and ranged from 1.23 to 27.78% with worm burdens between 2 and 609. *Nudacotyle novicia* also generally occurred at a low prevalence across the seven U.S. states where it has been reported (range: 0.4–23.85%) with worm burdens between 1 and over 700. *Q. quinqueserialis* had a consistently high prevalence across all studies and the highest worm burden of any trematode species ranging from 1 to 4,855 worms (60) (Table S1). High prevalence (>80%) of the trematodes *Plagiorchis nobeli* and *E. revolutum* has also been reported (Table S1).

The cause of death of one muskrat was attributed to severe liver infection of a trematode from the genus *Parametorchis* (50). This is the only account of any *Parametorchis* sp. found in muskrats. Aside from this individual case, no overt disease was reportedly associated with any of the other trematode infestations in muskrats.

#### Phylum Platyhelminthes: Class Cestoda: Order Cyclophyllidea

Cestodes (i.e., tapeworms) parasitize a diversity of aquatic and terrestrial species. Like trematodes, most cestodes have an indirect life cycle (61). Muskrats can serve as intermediate hosts for taeniid tapeworms (*Hydatigera taeniaeformis, Taenia* spp. and *Versteria mustelae*) and definitive hosts for several *Hymenolepis* species.

Cestodes were first identified in muskrats in the early 1900's (58). Since then, 40 articles spanning almost a century have reported at least 20 species of cestodes in muskrats from 17 U.S. states and seven Canadian provinces (Figure 1E). Since their first report, the taxonomic status of several muskrat parasites has changed either because they were reported prior to specific species being described (e.g., various *Cysticercus* spp.) or because of new genetic data (62). The new genetic data resulted in the reestablishment of the *Hydatigera* genus (in the case of the former *Taenia taeniaeformis, T. krepkogorski*, and *T. parva*), as well as the creation of the *Versteria* genus (formerly *Taenia mustelae*). The most commonly reported cestodes in muskrats as definitive hosts were *Hymenolepis* spp. (*n* = 32 studies) and *H. taeniaeformis* (*n* = 20 studies). *Hymenolepis evaginata* was the species reported most often (*n* = 21 studies).

The prevalence of cestodes in muskrat hosts has never exceeded 59% in any publication, when the number of individuals sampled was greater than one. Muskrats served as the definitive host for *Hymenolepis* spp. and prevalence ranged from 0 to 59%. The prevalence of *Hymenolepis* spp. was spatially explicit, with a higher prevalence occurring in northern North America (range: 26.19–58.82%), apart from 38.10% prevalence in Utah (63). Muskrats were the intermediate host for several taeniid species and burdens ranged from 1 to 15 worms and represented 83% of cases reporting debilitating cestode infections (*n* = 12). Intestinal cestode burdens rarely exceeded 30 worms; however, several cases of *Hymenolepis* spp. and one case of *Schizotaenia variabilis* exceeded 100 worms in the gastro-intestinal tract of individual muskrats (Table S1) (9, 64).

A 1956 study in Ohio documented a muskrat mortality due to severe liver infestation with *H. taeniaeformis* (reported as *Taenia taeniaeformis*) (65). In Poland, muskrats infected with the larval form of *H. taeniaeformis* were observed to have lower body mass as well as smaller body measurements (e.g., neck circumference) than uninfected muskrats (66). Recently, some species of *Taenia*, including one that infects muskrat, have been reclassified into the new genus *Versteria*. In 2016, researchers documented an introduced species of *Versteria* that caused a fatal infection in captive orangutan (*Pongo pygmaeus*) (67). During their investigation, they found many mustelids, including mink (*Neovison vison*), were definitive hosts for *Versteria* sp. This may be a cause of concern for muskrats since they share the same habitat as their main predator, mink. Two studies from North America documented muskrats as an intermediate host with liver cysts containing *V. mustelae* (reported as *T. mustelae*); however, genetic analysis was not conducted at the time and given recent taxonomic changes, molecular data are needed for species confirmation (63, 68). Regardless, this particular cestode was not reported as the cause of death in either study. Although mortality from *Versteria* sp. parasitism has not been reported in muskrats, the species has been reported in humans and further research is warranted (69, 70).

#### Phylum Nematoda

Phylum Nematoda (i.e., roundworms) consists of two classes, Chromadorea and Enoplea (71). Depending on the nematode species, transmission can occur through ingestion of intermediate hosts, through skin penetration, or orally by consuming food items (i.e., vegetation) contaminated by the eggs or larvae of a nematode species (72).

There are 36 articles reporting nematodes in muskrats dating back to 1915; however, very little contemporary data exist in peer-reviewed literature, with the latest article being from 1993 (73). Geographically, these articles represent 17 U.S. states and four Canadian provinces (Figure 1F). Nineteen species of nematodes have been reported from muskrats, and four of these species, *Hepaticola hepatica, Dirofilaria immitis, Capillaria michiganensis*, and *Baylisascaris procyonis* have been associated with disease (Table S1). The most commonly reported species are *Trichuris opaca* (*n* = 16) and *Calodium hepatica* (*n* = 10).

The prevalence of nematode parasites in muskrats exceeded 50% in only one study (47). *Capillaria* spp. had the highest prevalence in the literature (range: 0–61%) and worm burdens range from 1 to 692 worms. In 1981, a study reported the highest nematode burden of 692 specimens of *C. michiganensis* in a muskrat in Newfoundland (60). Prevalence of *T. opaca* ranged from 0.93 to 27.69% with burdens ranging from 1 to 103 worms (Table S1). Research conducted in 1946 and 1975 both included several samples from muskrats in Ottawa County, Ohio (74, 75). Differences in prevalence of *T. opaca* (1.43% in 1946 vs. 25% in 1975) suggest a possible increase in infection of this particular nematode in muskrats in Ottawa County. A contemporary study of *T. opaca* prevalence in muskrats from the same localities to determine if infection rates are increasing is warranted. There is a single adult-stage nematode associated with mortality in muskrats (22). Four muskrats from New York and one muskrat from Ontario reportedly died due to larval migrans caused by *B. procyonis* (22).

#### Phylum Acanthocephala

Acanthocephalans, also known as the “spiny-headed worms,” are parasites of the definitive host's intestinal tract (46). Six articles have reported two species of acanthocephalan in muskrats between the years 1947–1978, *Corynosoma* sp. and *Polymorphus* spp. (Table S1). Most reports of acanthocephalan in muskrats have been from two U.S. states and all provinces of Canada. Overall, the reported prevalence in these studies was low (<4%) with sample sizes exceeding 130 individuals (range: 130–326). Parasite burden ranged from 1 to 40, with the exception of a diagnostic report from Alberta where 138 *Polymorphus paradoxus* worms were collected from a single muskrat (76). That individual muskrat was the only reported case of the presence of all post larval stages of *P. paradoxus*. No other articles mentioned clinical signs in relation to acanthocephalan infestation.

#### Phylum Arthropoda: Subclass Pentastomida

Pentastomida parasites are crustaceans commonly known as “tongue worms” although most species reside in the respiratory system of their host (46). Pentastome infections are especially harmful to small mammals serving as the intermediate host when the infection intensity is high. Symptoms of pentastomiasis include abscesses, inflammation and granulomas (77).

Only two studies, both from Louisiana in the 1940's, reported a pentastomid (*Porocephalus crotali*) in muskrats (78, 79). Muskrats serve as intermediate hosts for *P. crotali* and infection was limited to adult animals located in scrub habitats (79, 80). The prevalence of *P. crotali* parasites in the two reports was low (9%) and parasite burden ranged between 1 and 1600 pentastomes (78, 79). One of the studies observed overt disease associated with pentastome infection in a single muskrat with over 1,600 nymphs embedded in all organs of the body (79). It is possible this parasite is more widespread in muskrats as the parasite has been reported in snake definitive hosts and other intermediate hosts (e.g., Virginia opossums (*Didelphis virginiana*), *Peromyscus* spp.) in the eastern U.S. (80, 81).

#### Phylum Arthropoda: Subclass Acari

Since 1936, ten articles have reported ten species of ectoparasites of muskrat from eight U.S. states and one Canadian province (Figure 1G). All ectoparasite species reported were mites with the exception of one flea species, *Orchopeas howardi* (*n* = 1 study) (82). The most commonly reported mite species were *Listrophorus* spp. (*n* = 7 studies), and *Laelaps multispinosa* (*n* = 7 studies) with parasite burdens ranging from 1 to >3000 and 0 to 811 mites, respectively. Other ectoparasite species reported include *Zibethicarus ondatrae* (*n* = 3 studies), *Myocoptes ondatrae* (*n* = 3 studies), *Radfordia zibethicalis* (*n* = 2 studies), *Androlaelaps fahrenholzi* (*n* = 1 study), *Labidophorus hypudai* (*n* = 1 study), *Myobia zibethicalis* (*n* = 1 study), *Schizocarpus indianensis* (*n* = 1 study), and an accidental finding of *Marsupialichus brasiliensis* (*n* = 1 study).

Most ectoparasite infestations in muskrats were not associated with overt disease (Table S1). A researcher in Illinois collected a muskrat that had an advanced myiasis: however, this may be secondary to trauma associated with trapping (83). A 2011 study conducted in Missouri compared percent body fat to the severity of ectoparasitic infestations (84). They found that individuals with an increasing intensity of *L. multispinosa* infestation (burden range: 1–42 mites) had a negative relationship with percent body fat. Considering reported *L. multispinosa* prevalence can range from 25 to 100% and parasite burdens can reach 811 mites per muskrat, this could be a cause of concern, particularly in the winter when fat reserves are crucial to a muskrat's survival.

### Toxins and Contaminants

Wildlife are exposed to a diversity of contaminants both naturally (e.g., heavy metal deposits, bioaccumulation) and unnaturally (i.e., anthropogenic means). Anthropogenic contaminants enter the environment through a variety of sources (e.g., wastewater, industrial discharge, lead ammunition, etc.) and can impact the health of humans, domestic animals, and wildlife (85, 86). Animals are exposed to environmental contamination, not only through consumption of contaminated waste and water, but also via consumption of plants and other food items that have absorbed contaminants. The ecology and foraging behaviors of the muskrat makes them particularly susceptible to exposure to environmental contamination in aquatic systems.

Muskrat exposure to contaminants has been reported in 12 papers representing seven U.S. states and three Canadian provinces (Figure 1H). With respect to other etiology reports, the investigations of muskrat exposure to contaminants are more recent, occurring between 1976 and 2014.

#### Heavy Metals

Heavy metals are dense metallic elements. Many of them (e.g., zinc, copper, and iron) are biologically important to the bodily function of many organisms, including humans. Other heavy metals (e.g., mercury and lead) do not hold any biological necessity to be absorbed into the body and can become toxic at specific concentration levels (87). Arsenic, cadmium, chromium, lead, and mercury are considered priority metals for surveillance due to their toxicity and potential effects on human and animal health (88). For most heavy metals, a variety of clinical syndromes occur at high exposure levels. The health impact of heavy metals at lower exposures is not sufficiently known (89).

Mercury (Hg) is not naturally found in organisms, and in its methylated forms bioaccumulates through the trophic levels. The range of lethal dose (LD_50_) for Hg reported for mammals is 10–40 μg/g (90). Although Hg poisoning is seemingly rare and mostly occurs in carnivores (i.e., domestic cat (*Felis catus*), ferret (*Mustela putorius furo*), mink, river otter (*Lontra canadensis*), there is potential that more cases could be found with increased surveillance since signs of poisoning (e.g., colic, dyspnea, etc.) are usually only noticed after chronic exposure (91, 92). No muskrats have been reported to have succumbed to Hg poisoning, but variable levels of Hg have been detected in individual muskrats (<0.01–0.69 μg/g) (92). During a study in Tennessee, researchers observed high Hg concentrations in hair samples from muskrats, however these muskrats were asymptomatic (Table 3) (96). This only occurred in adults at one out of the four sites sampled, with ranges in concentration for the combined remaining sites being low (0.03–1.07 μg/g). Hg concentrations in hair are known to be generally much higher than Hg concentrations in other tissues (i.e., liver or muscle) (97, 98).

**Table 3:** Mercury (Hg) concentrations found in muskrat (*Ondatra zibethicus*) tissue samples from four historical studies.

**Tissue sampled**	**Concentration (μg/g)**	***n***	**Location**	**References**
Kidney	0.011–0.019	76	Virginia	(93)
Liver	0.22	6	Washington	(94)
Liver	0.029–0.070	63	Pennsylvania	(95)
Hair	0.03–22.6	58	Tennessee	(96)

*Concentration levels have been converted to μg/g. For reference, LD_50_ for Hg ranges from 10–40 μg/g*.

Cadmium (Cd) can also has detrimental effects on animals and has been studied relatively extensively. Cadmium, a micronutrient, is absorbed by plants and animals and then is usually released back into the system through the excretion of urine and fecal matter (89). When Cd concentrations (animal LD_50_ = 225–890 μg/g) in the system build up, it can cause bone defects, myocardial disease, increase blood pressure, and affect DNA repair at the molecular level (99, 100). Cd toxicity was also reported as an immunosuppressant in mice as it decreased primary and secondary immune responses (101). Muskrats have not been reported to exhibit any detrimental effects of Cd exposure and have not had Cd concentrations higher than 0.32 μg/g in existing literature, which is much lower than the LD_50_ (Table 4).

**Table 4:** Cadmium (Cd) concentrations found in muskrat (*Ondatra zibethicus*) tissue samples from six historical studies.

**Tissue sampled**	**Concentration (μg/g)**	***n***	**Location**	**References**
Kidney	0.0008–0.0018	33	Ontario	(102)
Kidney	0.039–1.071	65	Pennsylvania	(95)
Kidney	0.08–3.08	76	Virginia	(93)
Kidney	0.11–0.157	126	Pennsylvania	(103)
Kidney	1.13	6	Washington	(94)
Liver	0.00025–0.00044	33	Ontario	(102)
Liver	0.0391–0.3157	65	Pennsylvania	(95)
Liver	0.042–0.064	126	Pennsylvania	(103)
None*	0.163	n/a	Montana	(104)

*Concentration levels have been converted into μg/g. For reference, the LD_50_ of Cd is 63-1125 μg/g*.

* *Cd concentration estimated using linear multimedia food-chain models based on ingestion rates for food items, soil, and water*.

Ingestion of environmental sources of lead (Pb) can result in toxicity. Lead poisoning in humans have been a well-studied disease for centuries (105). Lead toxicity is an important disease in multiple avian groups, including waterfowl through exposure to fishing tackle and ammunition in the environment and avian scavengers through exposure to ammunition in carcasses/tissues in game species (106–109). For mammals, lead poisoning has been reported in farm animals and can lead to a variety of syndromes (neurological, gastrointestinal, cardiovascular, etc.) (27, 110). Based on the habitat use of muskrats, they can be exposed to lead through a variety of sources, including road runoff and plant roots (111). Overt disease associated with lead toxicity has not been reported in muskrats; however, varying levels of exposure have been reported. The highest reported concentration of Pb in muskrats is 5.23 μg/g, which is just above the minimum level of toxicity in other rodent species (Table 5). A study conducted in Pennsylvania did observe that muskrats with higher Pb concentrations in their tissues were adults and came from marshes with high Pb concentrations in cattail tissues suggesting that lead is accumulated in muskrats over time through their food source (103).

**Table 5:** Lead (Pb) concentrations found in muskrat (*Ondatra zibethicus*) tissue samples from seven historical studies.

**Tissue sampled**	**Concentration (μg/g)**	***n***	**Location**	**References**
Kidney	0.0009–0.2689	64	Pennsylvania	(112)
Kidney	0.0032–0.0036	33	Ontario	(102)
Kidney	0.71–1.2	76	Virginia	(93)
Kidney	2.63–4.25	126	Pennsylvania	(103)
Liver	0.0020–0.0021	33	Ontario	(102)
Liver	0.0021–0.1537	64	Pennsylvania	(95)
Liver	0.27–0.96	6	Washington	(94)
Liver	3.71–5.23	126	Pennsylvania	(103)
Muscle	0.0–0.0048	64	Pennsylvania	(112)
Bone	1.117–2.226	64	Pennsylvania	(95)
n/a*	0	3	BC	(113)

*Concentration levels have been converted into μg/g. For reference, the minimum level of toxicity reported by the U.S. Environmental Protection Agency is 5μg/g*.

* *Tissue sampled was not noted, only that screening for various heavy metals did occur during the full necropsy*.

Arsenic (As) poisoning is not as common in wildlife as it is in humans. Exposure to inorganic As has been shown to cause birth defects in hamsters, especially those exposed to heat stress (114). Ronald Eisler from the U.S. Fish and Wildlife Service released a synoptic review of arsenic hazards to wildlife in 1988 (115). Many shorebirds and marine biota have arsenic concentrates, especially in tissues high in lipid content. Eisler reported that the LD_50_ for arsenic depends on species and ranges from 17 to 48 μg/g body weight and 2.5 to 33 μg/g body weight (bird and mammal, respectively). In aquatic systems, the LD_50_ varies depending on a variety of water properties (e.g., pH, temperature, etc.) Negative effects of As on aquatic species can occur with water concentrations anywhere between 19 and 48 μg As/l. Arsenic can be readily absorbed in the body of an organism and can decrease both white and red blood cell formation, and immune function, as well as cause brain damage and other physiological disturbances (89). Toxicity from As has not been reported in muskrats, but low levels of As have been detected in muskrat tissues (0.22 ppm) (Table S1).

#### Agricultural-Related Contaminants

Few studies (*n* = 2) have investigated exposure of muskrats to agriculture-related contaminants, such as pesticides, herbicides, and insecticides (Table 6). Together these studies screened muskrat tissue for eight contaminants including, atrazine, cyanazine, metolachlor (herbicides), chlorpyrifos, fonofos, terbufos (insecticides), Dichlorodiphenyldichloroethylene DDE (*p.p'*-DDE), and dieldrin. Only atrazine, dieldrin, and p.p'-DDE were identified at levels above the detection of these assays. No clear negative impacts were associated with the detection of these contaminants; however, muskrats in Virginia did have lower body condition associated with muskrats from one study area that was exposed to dieldrin (93).

**Table 6:** Agricultural-related contaminants and their concentrations found in muskrat (*Ondatra zibethicus*) tissues.

**Contaminant**	**Tissue sampled**	**Concentration (μg/g)**	***n***	**Location**	**References**
Atrazine	Subcutaneous fat	5.13–28.22	6	Illinois	(116)
Chlorpyrifos	Subcutaneous fat	0	6	Illinois	(116)
Cyanazine	Subcutaneous fat	0	6	Illinois	(116)
dieldrin	Liver and kidney	0.25	76	Virginia	(93)
Fonofos	Subcutaneous fat	0	6	Illinois	(116)
Metolachlor	Subcutaneous fat	0	6	Illinois	(116)
*p.p'*-DDE	Liver and kidney	0.03	76	Virginia	(93)
Terbufos	Subcutaneous fat	0	6	Illinois	(116)

*Concentration levels have been converted into μg/g*.

Although the influence of p.p.'DDE, dieldrin, and atrazine on muskrats is not defined, investigations in other mammals have been conducted. Studies on harbor seals (*Phoca vitulina*), sea lions (Ontariinae) and ringed seals (*Pusa hispida*) show that high levels of DDE were correlated with PCB (polychlorinated biphenyls) contamination and can negatively influence bodily function and reproductive success (117–119). The central nervous system and liver function are known to be affected by p.p'DDE contamination (120). Dieldrin is immunosuppressive and high levels of contamination in mice, birds, and other mammals is known to result in decreased lipid stores and death (121–123). Atrazine has been reported to cause increased mortality in frogs when co-contaminating an organism with other herbicides (124). Since many syndromes associated with contamination from agricultural compounds are vague and non-specific, and concentrations of these compounds have been detected in muskrats, further investigation on the impacts of agricultural contaminants on muskrats is warranted.

#### Other Contaminants

Concentration levels of two additional types of contaminants, PAHs (polycyclic aromatic hydrocarbons) and PCBs, have been reported in muskrat tissue samples. PAHs are chemicals found in a variety of products including coal tar, wood, and petroleum. Aerosol PAH contamination occurs when these products are burned, and soil and water contamination occur when the ashes are spread into the environment. Oil spills and aerial dispersal of coal dust can also result in environmental PAH contamination. PAHs are absorbed by plants and can be detected in plant tissue. Animals grazing on these plants can then accumulate PAHs in their tissues (125). Aquatic organisms are especially prone to PCB and PAH contamination, resulting in immunological and reproductive disorders (126). Halbrook et al. observed PAH concentrations in 22 of 35 muskrats at relatively low tissue concentrations between 0.03 and 0.15 ppm (93). The muskrats residing in sites with high total surface sediment PAH concentration had lower carcass and spleen weight, as well as lower fat indexes than the muskrats residing in low PAH concentrated areas, suggesting PAH contamination could be impacting muskrat health.

Several of PCB isomers are highly toxic to bodily functions and can result in immunotoxicity, weight loss, and dermal disorder, as well as other serious side-effects (127). In laboratory studies, levels of NOAEL (no-observed-adverse-effect-level) and LOAEL (lowest-observed-adverse-effect-level) for PCB's in mink livers were 2.03 μg/g lipid weight and 44.4 μg/g lipid weight, respectively (128). With increased bodily PCB concentration levels, reproductive toxicity is observed resulting in reduced relative litter size and kit survival (129). Historically, few studies have investigated PCB contamination in muskrats (*n* = 2). PCB concentration levels in 3.9% of liver and kidney samples (*n* = 76) were between 0.45 and 0.66 μg/g in Virginia (92). In the Hudson River Drainage, PCB concentrations in muskrat liver samples (*n* = 20) were up to 2.18 μg/g (130). No negative effects on muskrat health related to PCB concentration level was reported by either study.

## Discussion

As muskrat populations decline, it is critical we understand the possible role of disease, both historically and in the future. An important component of this understanding involves the characterization of pathogens, contaminants, and diseases that have been previously identified and monitoring changes over time. Historical reports have identified a number of pathogens or contaminants of potential concern for muskrat health. Notable parasitic diseases include coccidiosis and cysticercosis (22, 39). Ectoparasite infestation may have indirect impacts on muskrat health as higher infestations have been associated with a decreased in percent body fat (84).

Bacteria are the most important group of pathogens related to muskrat health and are the leading cause of muskrat mortality. Bacteria infecting muskrats can persist in the environment, resulting in outbreaks of disease that can decimate free-ranging muskrat populations returning to areas where the bacteria are present. There were six species of bacteria associated with muskrat mortality events; however, *F. tularensis, C. piliformis*, and cyanobacteria were the three associated with the highest individual mortality. With only one report in the U.S., little is understood or documented about the impacts of cyanobacteria on muskrats. Many of the reports of bacterial infections from the species above and viral infections were only documented after outbreaks occurred. The prevalence of these infections in the outwardly healthy muskrat population is poorly documented, so there is an unclear understanding of the risk of infection via these bacterial species or viruses.

There is little noted about acanthocephalan parasites in muskrats; however, infection by acanthocephalan species has been documented to cause mortality in juvenile sea otters and might be a source of interest in future muskrat studies. *Dracunculus* spp. also cause severe infections in other wildlife species and was documented in muskrats in the 1970's but has not been noted in the literature since. Unlike other parasites of muskrats, *Dracunculus* spp. are found in the subcutaneous regions of the extremities and may go unobserved during traditional parasitic surveys of the body cavity. The unicellular parasitic eukaryote *E. cuniculi* has been documented in muskrats and has been another notable cause of wildlife mortality that should be investigated further in muskrats (131).

There is a need for further research on the effects of contaminants on mammal health, especially where muskrats are involved. Muskrats exist at the mid-trophic level, are semi-abundant, and live in aquatic environments that serve as reservoirs for high concentrations of many environmental contaminants. They are a prime study species for understanding the impacts of environmental contamination in ecological systems because they can bioaccumulate contaminants from their food source, and they influence the bioaccumulation of contaminants of other organisms at higher-trophic levels. Without knowing what bodily contamination level is toxic to muskrats for various chemicals and heavy metals, it is difficult to determine their effects on individual muskrats, let alone population dynamics. Also, there were only two studies conducted on contamination levels of agricultural-related contaminants in muskrat tissues, and only a few reports on PAH and PCB levels in muskrats and their influences. However, in the few studies conducted on PAH and PCB levels in muskrat tissues, the authors noted effects on body condition and reproduction, which merits further research on the topic.

Muskrats serve as sentinel species for many pathogens and diseases, including *Giardia* spp., *Cryptosporidium* spp., *D. insignis*, and echinostomes. They can also be used as sentinels for environmental contamination to assess aquatic ecosystem health. Continued or sustained monitoring of muskrat health parameters can help determine human health risks as many of the pathogens and contaminants that muskrats harbor have health impacts for domestic animals and humans. There is still much that is not well-understood about the health of muskrat populations and the influences of disease, parasites and contaminants on survival. The intent of this paper is that it be used as a reference for future investigations on ways to build upon previous research. There are gaps in the knowledge of contaminant toxicity and bacterial prevalence, and much of the geographic distribution of pathogens and disease vectors of muskrats have yet to be documented. Continued active and passive surveillance for these pathogens and vectors, as well as for new ones that may emerge or be detected using new techniques, is encouraged.

## Author Contributions

LG, JB, ML, and WW provided substantial contributions to the conception and design of the review. LG did the literature review and writing of initial draft and revisions. JB and MY provided expertise on subject, crucial interpretation of data, and critically revised the manuscript for important intellectual content. JB, ML, and WW provided commentary and final review of drafts.

## Conflict of Interest

The authors declare that the research was conducted in the absence of any commercial or financial relationships that could be construed as a potential conflict of interest.

## Supplementary Material

Table S1 Compilation of reports of muskrat exposure to pathogens, toxins and contaminants organized by the following subjects: Viruses, Bacteria, Parasites, Contaminants, and Other.
